# Dual-task related gait changes after CSF tapping: a new way to identify idiopathic normal pressure hydrocephalus

**DOI:** 10.1186/1743-0003-10-117

**Published:** 2013-12-21

**Authors:** Gilles Allali, Magali Laidet, Olivier Beauchet, Francois R Herrmann, Frederic Assal, Stephane Armand

**Affiliations:** 1 Department of Clinical Neurosciences, Division of Neurology, Geneva University Hospitals and University of Geneva, 4 rue Gabrielle-Perret-Gentil, Geneva 1211, Switzerland; 2 Department of Neurology, Albert Einstein College of Medicine, Yeshiva University, Bronx, New York, USA; 3 Department of Neuroscience, Division of Geriatrics, Angers University Hospital; UPRES EA 2646, University of Angers, UNAM, 4 Boulevard de Lavoisier, Angers 49000, France; 4 Department of Internal medicine, Rehabilitation and Geriatrics, Geneva University Hospitals and University of Geneva, Geneva, Switzerland; 5 Willy Taillard Laboratory of Kinesiology, Geneva University Hospitals and University of Geneva, Geneva, Switzerland

**Keywords:** Gait disorders, Idiopathic normal pressure hydrocephalus, Dual tasking, Executive function, Cerebrospinal fluid

## Abstract

**Background:**

Gait disturbances found in patients with idiopathic normal pressure hydrocephalus (iNPH) are unspecific to the diagnosis and commonly occur in neurodegenerative or vascular conditions (iNPH-like conditions). This current retrospective pre-post intervention study aims to determine whether changes in quantitative gait parameters during dual task condition differed between iNPH and iNPH-like conditions before and after cerebrospinal fluid (CSF) tapping.

**Methods:**

49 patients assessed before and after CSF tapping were included in this study (27 with iNPH and 22 with iNPH-like conditions). Gait analysis during single and dual task conditions (walking and backward counting) was performed before and after a CSF spinal tap of 40 ml. Gait parameters were compared between iNPH and iNPH-like conditions patients. Logistic regressions were used to examine the association between iNPH and gait parameters.

**Results:**

Improvements of step width (−9.03 (20.75)% for iNPH group; +0.28 (21.76)% for iNPH-like conditions group), stride length (+7.82 (20.71)% for iNPH group; -0.62 (19.22)% for iNPH-like conditions group), walking speed (+12.20 (29.79)% for iNPH group; +2.38 (32.50)% for iNPH-like conditions group) and stance duration (−1.23 (4.03)% for iNPH group; +0.49 (5.12)% for iNPH-like conditions group) during dual task, after CSF spinal tapping, were significant in patients with iNPH compared to patients with iNPH-like conditions. No between group difference was observed for the single walking task evaluation. The multiple logistic regression revealed that among these four gait parameters, only the improvement in step width was associated with the diagnosis of iNPH.

**Conclusion:**

Dual-task related changes in spatio-temporal gait parameters before and after CSF tapping might be a novel and discriminative method of identifying iNPH patients from other similar conditions.

## Introduction

Idiopathic normal pressure hydrocephalus (iNPH) was first identified by Salomon Hakim in 1957. It is a communicating hydrocephalus characterized by enlarged ventricles visible on brain imagery and its clinical presentation relies on a triad of symptoms affecting gait, cognition and urinary incontinence. Gait difficulties are usually the first symptoms of the disease that appear insidiously between the sixth and eighth decade of life and include an apraxic, glue-footed, magnetic or parkinsonian gait [1]. The uncertainty surrounding diagnosis of iNPH patients is particularly problematic, because symptoms of iNPH are unspecific. Identifying patients with iNPH from other patients with higher level gait disorders, vascular dementia or even Parkinson’s disease remains a real challenge for clinicians. If quantitative gait analysis has revealed a decreased stride length, decreased foot-to-foot clearance and a broad-based gait in iNPH patients compared to age-matched healthy controls [2], there is an urgent need to find new markers that might better differentiate among closely related gait conditions and aid therapeutic decisions, i.e. neurosurgical shunt placement.

Dual-task related gait changes refer to any modification when walking while simultaneously performing an attention-demanding task and represent an interesting paradigm to assess in parallel, gait and cognitive functions, which are both deficient in iNPH. These changes are related to the capacity to share attention between the two tasks, and strongly depend on executive functions [3]. Cognitive deficits in iNPH typically involve executive functions and are potentially improved after shunt placement [4]. Our recent study demonstrated that the dual-task paradigm was a good marker of gait improvement after cerebrospinal fluid (CSF) tapping in a clinical sample of patients with iNPH [5].

This study aims to compare spatio-temporal gait parameters before and after CSF tapping under single and dual task conditions in patients with iNPH and in patients with other gait disorders mimicking this pathology. Since it is known that spinal tapping improves gait in iNPH, and that dual-tasking better reveals gait improvement, we further hypothesize that patients with iNPH will present with increased gait changes under dual task conditions after CSF tapping in comparison with patients with iNPH-like conditions.

## Methods

### Participants

**Figure 1:**
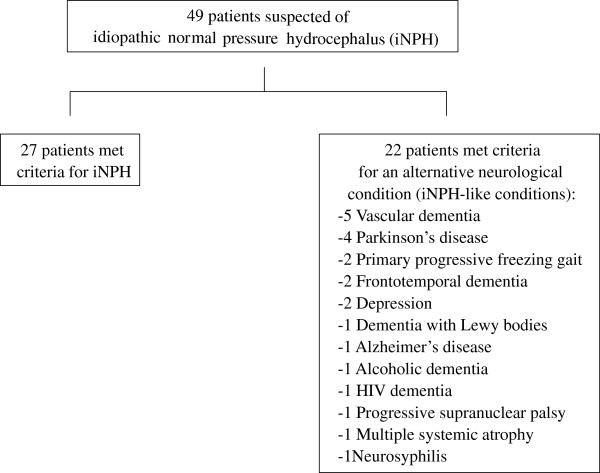
Study profile.

A total of 49 patients suspected of iNPH at the Department of Neurology at the Geneva University Hospitals were included in this study: twenty-seven patients fulfilled the iNPH consensus guideline criteria [6] and twenty-two patients presented with an alternative neurological diagnosis (age median (IQR); 77.0 (10.0) years and 74.5 (9.0) years respectively; p-value: 0.62) (Table 1): vascular dementia (five patients), Parkinson’s disease (four), primary progressive freezing gait (two), Frontotemporal lobar degeneration (two), depression (two), dementia with Lewy bodies (one), Alzheimer’s disease (one), alcoholic dementia (one), HIV dementia (one), progressive supranuclear palsy (one), multiple system atrophy (one) and neurosyphilis (one) (Figure 1). The patients gait was analyzed twice, before and then after CSF tapping of 40 ml [1] (time between CSF tapping and second gait evaluation: 2.10 (1.49) days). Exclusion criteria included: acute medical illness in the past three months, orthopedic or rheumatologic disorders interfering with gait, patients receiving CSF tapping in the 3 months preceding the assessment, a change in the treatment between the two gait assessments, unable to walk a minimum of 15 m without a walking aid and not able to perform the dual task evaluation (walking while backwards counting).

**Table 1:** Clinical characteristics of subjects (n = 49)

	**iNPH (n = 27)**	**iNPH-like conditions (n = 22)**	P-value*
Age (years)	77.0 (10.0)	74.5 (9.0)	0.62
Female, n [%]	10 [37]	6 [27]	0.55
Disease duration (months)	30.0 (36.0) ‡	24.0 (39.0) ≈	0.91
Comorbidities (n)	4.0 (4.0)	5.00 (3.0)	0.15
Treatments (n)	5.0 (3.0)	5.5 (5.0)	0.28
Psychoactives drugs (n)	1.0 (1.0)	1.0 (2.0)	0.50
iNPH grading scale^¥^ (n)			
Gait disturbance (/4)	2 (0.0)	2 (0.0)	0.76
Cognitive impairment (/4)	2 (1.0)	2 (0.0)	0.20
Urinary disturbance (/4)	2 (2.0)	0 (1.5) ≈	**0.04**

iNPH, idiopathic normal pressure hydrocephalus.

Values are medians (interquartile ranges).

Percentages are indicated in brackets.

‡Based on 24 subjects.

≈Based on 20 subjects.

¥Ranges from 0 to 4, with higher scores indicating worse symptoms [7].

*Comparison based on Mann–Whitney test or Fisher exact test as appropriate.

Significant differences (p-values) are highlighted in bold characters.

Gait disorders, cognitive impairment and urinary disturbance were graded using the iNPH grading scale [7]. The iNPH grading scale is used to separately evaluate the severity of each of the three disorders. The score of each domain ranges from 0 to 4 with higher scores indicating worse symptoms. Among the iNPH patients group, nine patients accepted the surgical procedure (ventriculo-peritoneal shunt).

### Gait recordings

Synchronized footswitches (AURION ZeroWire, Milan, Italy, sampling rate of 1000 Hz) and a seven-camera opto-electronic system (VICON Mx3+, Vicon Motion Systems, Oxford, UK, sampling rate of 100 Hz) were used. The 3D position of two reflective markers placed on the foot (on both heels and both 2nd metatarsals) and the temporal data of the footswitches were combined to compute gait parameters including walking speed, stride length, stride time, stance duration (measured as the percentage of the gait cycle), step width and step height (maximum distance between the heel marker and the floor during the swing phase on the vertical axis minus the mean position of the heel marker during mid-stance). These gait parameters were assessed whilst walking at a self selected walking speed on a 10-m walkway in single-task and dual-task (backward counting aloud by subtracting serial 1 from 50) conditions in a random order. The number of figures enumerated while walking was taken into account. To compare the inter-subject differences, the number of enumerated figures during the walking time was calculated in minutes. Before testing, a trained evaluator gave standardized verbal instructions on the test procedure. For the dual task condition, patients were asked to walk and to count backwards to the best of their ability without any task prioritization. The difference of gait parameters and cognitive performances between before and after CSF tapping was calculated according to the following formula: [(Performance after CSF tapping – performance before CSF tapping)/(performance before CSF tapping)] × 100.

### Statistics

The distribution of gait parameters was not gaussian even after a trial of normalization. The normality of the parameters’ distribution was checked with Shapira-Francia tests. Therefore, non-parametric tests were performed and medians, along with interquartile ranges (IQR) were reported. First, between-groups comparisons were performed using Mann–Whitney test, or Fisher exact test, as appropriate. Secondly, univariate (model 1), and multiple logistic regressions (model 2) were used to examine the association between iNPH (independent variable) and gait parameters during dual task conditions. *P*-values less than 0.05 were considered statistically significant. The pseudo R square is to logistic regression what the R square is to linear regression that is the coefficient of determination, which corresponds to the amount of variance explained by the model. All statistics were performed using the Stata Statistical Software, version 12.0.

### Standard protocol approvals, registrations, and patient consents

This retrospective study protocol was approved by the ethical committee at Geneva University Hospitals. All subjects gave informed consent according to the ethical standards set forth in the declaration of Helsinki (1983).

## Results

Demographic and clinical characteristics are summarized in Table 1. Both groups presented the same clinical characteristics, except in terms of incontinence, which was more severe in the iNPH group (iNPH grading scale [7] incontinence (/4): 2.0 (2.0) and 0.0 (1.5)) (Table 1). Before CSF tapping, no statistical difference between the two groups was observed for any of the measured gait parameters during single and dual task conditions. For the cognitive performance of the dual task, no statistical difference was found between the two groups.

No statistical difference between the two groups was observed for the delta of the gait parameters under single task condition (Table 2). Under dual-task conditions, the delta of the walking speed, stride length, step width and stance duration was significantly improved in the iNPH group, meaning that (i) iNPH patients increased their walking speed between the pre and post- CSF tapping more than the iNPH-like conditions group (+12.20 (29.79) and +2.38 (32.50)% respectively) (Table 2); (ii) iNPH patients increased their stride length between the two evaluations more than the iNPH-like conditions group (+7.82 (20.71) and −0.62 (19.22)% respectively) (Table 2); (iii) iNPH patients decreased their step width between the two evaluations more than the iNPH-like conditions group (−9.03 (20.75) and +0.28 (21.76)% respectively) (Table 2); and iNPH patients decreased their stance duration between the two evaluations more than the iNPH-like conditions group (−1.23 (4.03)% and +0.49 (5.12)% respectively) (Table 2). For the cognitive component of the dual task, the delta of the cognitive task was identical between the two groups.

**Table 2:** Clinical performance of subjects (n = 49) and comparison of delta performances¶ between iNPH and iNPH-like conditions

	**iNPH**		**iNPH-like conditions**		P-value*
	**(n = 27)**		**(n = 22)**		
	**Pre-LP**	**Post-LP**	**Pre-LP**	**Post-LP**	
Single task gait parameters					
Walking speed (m/s)	0.65 (0.37)	0.78 (0.24)	0.66 (0.46)	0.68 (0.42)	
Delta^¶^ (%)	+11.15 (22.28)		+4.23 (14.82)		0.054
Stride time (s)	1.21 (0.23)	1.19 (0.19)	1.22 (0.20)	1.21 (0.24)	
Delta^¶^ (%)	−4.09 (15.07)		+0.58 (8.34)		0.278
Stride length (m)	0.88 (0.40)	0.93 (0.28)	0.80 (0.43)	0.80 (0.36)	
Delta^¶^ (%)	+7.51 (18.67)		+0.20 (10.39)		0.077
Step width (m)	0.10 (0.09)	0.10 (0.05)	0.09 (0.05)	0.10 (0.06)	
Delta^¶^ (%)	−7.70 (34.12)		+3.54 (43.71)		0.091
Step height (m)	0.17 (0.06)	0.20 (0.06)	0.17 (0.08)	0.18 (0.07)	
Delta^¶^ (%)	+4.47 (11.21)		+1.36 (9.12)		0.056
Stance duration**	66.70 (3.43)	66.22 (4.60)	66.10 (7.00)	66.41 (6.14)	
Delta^¶^ (%)	−1.03 (6.69)		−0.32 (7.11)		0.553
Cognitive component					
Backward counting	43.24 (36.82)	52.44 (46.69)	59.81 (42.31)	63.72 (36.65)	
Delta^¶^ (%)	+15.98 (30.56)		+4.75 (46.28)		0.101
Dual task‡ gait parameters					
Walking speed (m/s)	0.54 (0.39)	0.64 (0.36)	0.46 (0.37)	0.56 (0.32)	
Delta^¶^ (%)	+12.20 (29.79)		+2.38 (32.50)		**0.044**
Stride time (s)	1.37 (0.30)	1.26 (0.22)	1.34 (0.39)	1.32 (0.31)	
Delta^¶^ (%)	−6.33 (16.03)		−1.17 (11.47)		0.148
Stride length (m)	0.76 (0.38)	0.83 (0.40)	0.67 (0.45)	0.74 (0.38)	
Delta^¶^ (%)	+7.82 (20.71)		−0.62 (19.22)		**0.030**
Step width (m)	0.12(0.08)	0.11 (0.08)	0.10 (0.08)	0.09 (0.06)	
Delta^¶^ (%)	−9.03 (20.75)		+0.28 (21.76)		**0.009**
Step height (m)	0.17 (0.07)	0.18 (0.06)	0.16 (0.07)	0.17 (0.07)	
Delta^¶^ (%)	+5.20 (10.55)		+0.40 (9.76)		0.051
Stance duration**	68.48 (4.86)	67.16 (5.13)	67.83 (7.72)	68.74 (6.61)	
Delta^¶^ (%)	−1.23 (4.03)		+0.49 (5.12)		**0.047**
Cognitive component					
Backward counting	45.44 (36.60)	47.44 (42.20)	53.63 (39.23)	61.37 (38.24)	
Delta^¶^ (%)	+21.02 (53.02)		+0.60 (41.48)		0.294

iNPH, idiopathic normal pressure hydrocephalus.

LP, lumbar puncture.

Backward counting correspond to the number of enumerated figures.

Values are medians (interquartile ranges).

^¶^Calculated according to the following formula: [(performance after LP – performance before LP)/(performance before LP)] × 100.

*Comparison based on Mann–Whitney test. Significant differences (p-values) are highlighted in bold characters. P-values are based on the comparison of the delta between the iNPH group and the iNPH-like conditions group.

**Stance duration are presented as a percentage of the gait cycle.

‡Gait while backward counting.

Under dual-task, univariate (model 1) and multiple logistic regression (model 2) showed that among the gait parameters, only the delta of step width was associated with the group of iNPH (Table 3).

**Table 3:** Univariate (model 1) and multiple logistic regressions (model 2) showing an association between iNPH (independent variable) and gait parameters during delta* of dual task (dependant variable)

	**Model 1 (nonadjusted)**					**Model 2 (adjusted)**		
	Odds ratio	95% CI	P-value	r^2^	Odds ratio	95% CI	P-value	r^2^
Step width	0.95	[0.92; 0.99]	**0.017**	0.104	0.95	[0.91; 0.99]	**0.020**	0.141
Stride length	1.02	[0.99; 1.04]	0.185	0.042	0.97	[0.91; 1.03]	0.318	
Walking speed	1.02	[1.00; 1.04]	0.104	0.059	81.03	[0.25; 25922.77]	0.135	
Stance duration	0.92	[0.83; 1.03]	0.146	0.036	1.01	[0.88; 1.16]	0.901	

iNPH, idiopathic normal pressure hydrocephalus.

CI,Confidence interval.

Significant differences (p-values) are highlighted in bold characters.

*Calculated according to the following formula: [(performance after LP – performance before LP)/(performance before LP)] × 100.

r^2^, R square.

## Discussion

In this study, we evaluated the use of quantitative spatio-temporal gait parameters under dual task conditions before and after CSF tapping for the diagnosis of iNPH in patients with a suspicion of iNPH. As hypothesized, the improvement of step width, stride length, walking speed and stance duration whilst walking under dual task conditions after CSF tapping was significantly better in iNPH patients than in iNPH-like conditions. Among these four gait parameters, step width improvement after CSF tapping during dual task seems to be the most discriminative parameter. Interestingly, the discriminative features of gait parameters between iNPH and iNPH-like conditions were observed only for dual task and not for usual single task gait assessment.

iNPH symptoms and typically gait disorders are not specific, occurring in many neurological conditions, like those presented in the iNPH-like conditions group (i.e. Parkinson’s disease, vascular dementia). A previous comparative analysis of gait parameters in individuals with iNPH and Parkinson’s disease revealed that the gait pattern of iNPH was clearly distinguishable from that of individuals with Parkinson’s disease: this was demonstrated by an improvement in Parkinson’s disease due to external cues, and an increased step width in iNPH that was shown to be a critical marker of iNPH [8]. In clinical practice, physicians need to identify iNPH from other undefined medical conditions, tests such as CSF tapping can aid diagnosis, although this is not included in the iNPH consensus guidelines [6]. Interestingly, from a clinical perspective, patients with iNPH have a tendency to fall backwards and as compensation, a broad-based gait is employed by the patients to increase their stability. This specific improvement of stride width after CSF tapping could be due to the combined effect of stability and gait. Previous studies have shown that symptomatic improvements after CSF tapping can increase the likelihood of a favourable response to a shunt [9]. However, Ondo et al. showed that 37.5% of patients with vascular parkinsonism also reported a significant gait improvement after CSF tapping [10]. Ondo et al. evaluated gait improvement two months after spinal fluid removal, assessed by a subjective auto-evaluation, and using a standard single task gait evaluation. Indeed, in a recent retrospective study, none of the patients that underwent an invasive diagnostic procedure for suspected iNPH, and that presented with an alternative neurological diagnosis after shunt placement experienced definite improvement in any symptom three years post neurosurgery [11]. This study of the cognitive component of gait (i.e. dual task) before and after CSF tapping could represent an additional gait marker of iNPH prior to shunting.

Dual-task-related gait changes reflect, in part, the influence of cognitive functions on gait, and in particular, executive functions. Indeed, the ability to dual-task requires an intact capacity to appropriately allocate attention between two tasks performed simultaneously. Pathological interference of a cognitive task while walking has been shown in different neurological conditions, such as Parkinson’s disease [12], vascular dementia [13], behavioural variant of frontotemporal dementia [14] and Alzheimer’s disease [15]. These conditions share a similar neuropsychological profile with iNPH. As well as gait improvement, previous studies have shown an improvement in executive functioning and attention after shunt placement in iNPH [11,16]. The improvement of gait parameters during dual task (step width, step length, stance duration and walking speed) after CSF tapping showed in iNPH patients indicates a better capacity to specifically allocate attention to gait, and not to the cognitive component of the dual task. The pressure of the distended ventricles on critical cerebral sites in iNPH might be a potential pathophysiological explanation [2]. Following CSF tapping, periventricular regional pressure modification would positively influence the frontosubcortical circuits involved in the dual-task-related gait changes. Simultaneous assessment of gait and cognition using dual-task may better reflect the potential benefits of CSF tapping than a separate evaluation of gait and cognition in iNPH patients.

The main limitations of our study include a lack of autopsy-confirmed diagnosis, a small sample size and the specificity of the exclusion criteria; i.e. patients that were unable to walk without a walking aid. Additionally, adding an older control group would be very interesting to better understand the effect of CSF tapping on gait parameters during dual task in healthy individuals. Finally, the patient’s cognitive performances combined with the dual-task gait approach should be assessed in more detail in a further prospective design study.

## Conclusion

Patients with iNPH present with a reduced step width, an increased walking speed, an increased stride length and a decreased stance duration while walking under dual task conditions after CSF tapping in comparison with patients with iNPH-like conditions. The dual task paradigm represents a simple and easy approach to combine the evaluation of gait and cognition simultaneously; both are known to be independently improved by CSF tapping. These results suggest that combining quantitative gait assessment during dual task conditions after CSF tapping could improve the clinical evaluation of patients with a suspicion of iNPH prior to shunting.

## Abbreviations

CSF: Cerebrospinal fluid; iNPH: Idiopathic normal pressure hydrocephalus; LP: Lumbar puncture.

## Competing interests

The authors declare that they have no competing interests.

## Authors’ contributions

GA has full access to the data in the study and takes responsibility for the integrity of the data and the accuracy of the data analysis. Study concept and design: GA and FA; Acquisition of data: ML and SA; Analysis and interpretation of data: GA, ML, OB, FRH, FA, SA; Drafting of the manuscript: GA; Critical revision of the manuscript: ML, OB, FRH, FA, SA; Obtained funding: GA; Statistical expertise: FRH; Administrative, technical and material support: ML and SA; Study supervision: GA and FA. All authors read and approved the final manuscript.
